# Simulation and qualitative analysis of glucose variability, mean glucose, and hypoglycemia after subcutaneous insulin therapy for stress hyperglycemia

**DOI:** 10.1186/s12976-016-0029-2

**Published:** 2016-01-27

**Authors:** Richard J. Strilka, Mamie C. Stull, Michael S. Clemens, Stewart C. McCaver, Scott B. Armen

**Affiliations:** Department of Trauma and Critical Care Surgery, San Antonio Military Medical Center, 3551 Roger Brooke Drive, Fort Sam Houston, San Antonio, TX USA; Walter Reed National Military Medical Center, 8901 Rockville Pike, Bethesda, MD USA; Division of Trauma, Acute Care and Critical Care Surgery, Pennsylvania State College of Medicine, 500 University Drive, Hershey, PA USA

**Keywords:** Stress hyperglycemia, Subcutaneous insulin, Glucose variability, Mean glucose, Hypoglycemia, Computer simulation

## Abstract

**Background:**

The critically ill can have persistent dysglycemia during the “subacute” recovery phase of their illness because of altered gene expression; it is also not uncommon for these patients to receive continuous enteral nutrition during this time. The optimal short-acting subcutaneous insulin therapy that should be used in this clinical scenario, however, is unknown. Our aim was to conduct a qualitative numerical study of the glucose-insulin dynamics within this patient population to answer the above question. This analysis may help clinicians design a relevant clinical trial.

**Methods:**

Eight virtual patients with stress hyperglycemia were simulated by means of a mathematical model. Each virtual patient had a different combination of insulin resistance and insulin deficiency that defined their unique stress hyperglycemia state; the rate of gluconeogenesis was also doubled. The patients received 25 injections of subcutaneous regular or Lispro insulin (0-6 U) with 3 rates of continuous nutrition. The main outcome measurements were the change in mean glucose concentration, the change in glucose variability, and hypoglycemic episodes. These end points were interpreted by how the ultradian oscillations of glucose concentration were affected by each insulin preparation.

**Results:**

Subcutaneous regular insulin lowered both mean glucose concentrations and glucose variability in a linear fashion. No hypoglycemic episodes were noted. Although subcutaneous Lispro insulin lowered mean glucose concentrations, glucose variability increased in a nonlinear fashion. In patients with high insulin resistance and nutrition at goal, *“rebound hyperglycemia”* was noted after the insulin analog was rapidly metabolized. When the nutritional source was removed, hypoglycemia tended to occur at higher Lispro insulin doses. Finally, patients with severe insulin resistance seemed the most sensitive to insulin concentration changes.

**Conclusions:**

Subcutaneous regular insulin consistently lowered mean glucose concentrations and glucose variability; its linear dose-response curve rendered the preparation better suited for a sliding-scale protocol. The longer duration of action of subcutaneous regular insulin resulted in better glycemic-control metrics for patients who were continuously postprandial. Clinical trials are needed to examine whether these numerical results represent the glucose-insulin dynamics that occur in intensive care units; if present, their clinical effects should be evaluated.

## Background

Stress hyperglycemia (SH) of critical illness is a multifaceted disease that involves elevated hepatic gluconeogenesis [1–3], increased insulin resistance (*IR*) [4–9], and insulin deficiency (*ID*) [5, 10]. These factors are dynamic and evolve during critical illness. In combination, they produce a heterogeneous disorder marked by hyperglycemia that affects 30–50 % of intensive-care patients although some may have occult diabetes [11–13]. Optimal SH therapy would minimize glucose variability (*GV*) [14–16], prevent hyperglycemia [17, 18], and not cause hypoglycemia [19, 20] because all three factors have a similar mortality risk [21].

Literature review suggests that patients with SH have variable responses to the same insulin treatment [16]. Disease heterogeneity may partially explain why only certain subgroups, such as trauma patients, seem to benefit from tight glycemic control (TGC) [22, 23]. In general, TGC studies have been plagued by frequent hypoglycemia [22, 24] and worsened mortality [25]. One current treatment recommendation for SH is to begin therapy with an insulin infusion when glucose concentrations exceed 150 mg/dL, with an absolute goal of less than 180 mg/dL [16]. As the patient recovers, the insulin infusion is changed to a subcutaneous (SQ) insulin sliding scale after vasopressors have been removed, peripheral edema has resolved, and no further nutrition interruptions have been planned. Nevertheless, SH may persist for weeks because of altered gene expression that results in long-term changes in glucose metabolism [26]. Therefore, an improved understanding of SQ insulin therapies may benefit critically ill patients in the subacute phase of their illness. Although SQ insulin therapy has been successfully used in critical care [27, 28], the optimal short-acting insulin preparation is unknown; moreover, *GV* after SQ insulin therapy has not been examined.

SQ Lispro insulin improves mean glucose concentration (*MGC*) in diabetes while minimizing postprandial hypoglycemia [29, 30]. When an otherwise healthy diabetic patient eats a meal, endogenous insulin concentrations spike and follow a narrow “bell shape” curve [31]. Clinical [32] and computational studies [33] confirm that SQ Lispro insulin tends to outperform SQ regular insulin in this patient population because of the shorter duration of action that more closely mimics the body’s response to a food bolus. On the other hand, patients with SH who are receiving continuous nutrition may have a very different response to short-acting insulin preparations, given that they are continually postprandial. Assuming stable values for *IR*, *ID*, gluconeogenesis and the continuous nutrition rate, their hyperglycemia would result from a uniformly amplified glucose ultradian oscillation.

We hypothesized that the short-acting SQ insulin preparation with the longer duration of action would have a better *GV* profile, and a smaller risk of hypoglycemia, in the background of continuous enteral feedings when SH is present. Thus, our aim was to conduct a qualitative computational study of the glucose-insulin axis to simulate a population of critically ill patients with SH to identify the optimal short-acting SQ insulin therapy that should be used in this clinical scenario. In addition, the effects of SH disease heterogeneity on the patients’ response to different short-acting SQ insulin therapies were also examined.

## Methods

In the last decades, several mathematical models have been proposed and studied with the aim of better understanding the dynamics of the glucose-insulin axis, so that safer and more effective insulin administration practices could be developed to treat diabetes mellitus [33–39]. This work has meet with success and has culminated in a recent clinical trial of an artificial pancreas [40]. These analytical methods can be modified to model the effects of SQ insulin when used against SH in the Intensive Care Unit [41]. The analysis will be qualitative because the model used has not been validated against human data gathered from the critically ill; however, closely related models have reproduced results from clinical trials involving diabetes and SQ insulin therapies [33, 42].

### The glucose-insulin system in functional form

The model used in this study [38, 41] applies delay differential equations to incorporate the time delays required to simulate the finite response time of the pancreas and liver to secrete insulin and glucose, respectively. The delay differential equations were derived from the principle of mass conservation for both the glucose concentration, *G*(*t*), and the insulin concentration, *I*(*t*), for any time t. The principle states that the rate of change in *G*(*t*) must equal the glucose production, *G*_*p*_(*t*), minus glucose utilization, *G*_*u*_(*t*). Similarly, the rate of change of *I*(*t*) must equal the insulin production, *I*_*p*_(*t*), minus insulin clearance, *I*_*c*_(*t*). In equation format, this principle reads as follows:

(2.1a) $$\begin{array}{*{20}l}  { {d G(t)} \over {dt}} =& G_{p}(t) - G_{u}(t), \end{array} $$

(2.1b) $$\begin{array}{*{20}l}  { {d I(t)} \over {dt}} =& I_{p}(t) - I_{c}(t), \end{array} $$

where

(2.2a) $$\begin{array}{*{20}l} & {G_{p}(t)} = { G_{in} (t) + \boldsymbol{\digamma} \times f_{5}(I(t-\boldsymbol{\tau_{2}})) \times f_{6}(G(t)) },  \end{array} $$

(2.2b) $$\begin{array}{*{20}l}  & {G_{u}(t)} = { f_{2}(G(t)) + \boldsymbol{\beta} \times f_{3}(G(t)) \times f_{4}(I(t))+ f_{7}(G(t)-330) }, \end{array} $$

(2.2c) $$\begin{array}{*{20}l}  & {I_{p}(t)} = {I_{in}(t) + \boldsymbol{\alpha} \times f_{1}(G(t-\boldsymbol{\tau_{1}}))}, \end{array} $$

(2.2d) $$\begin{array}{*{20}l}  & {I_{c}(t)} = {\boldsymbol{d_{i}} I(t)}, \end{array} $$

and ${{d}\over {dt}}$ is the derivative with respect to time. The functions *f*_1_ through *f*_7_ are highly nonlinear and describe different characteristics of the glucose-insulin axis; their explicit forms are presented below. They describe the body’s glucose production and utilization as well as insulin production and clearance. Note that *G*_*in*_(*t*) in Eq. (2.2a) denotes glucose absorption from either enteral nutrition or an intravenous source. Insulin absorption from an exogenous source is represented by *I*_*in*_(*t*) in Eq. (2.2c); this function will be used to represent the different SQ insulin therapies. The purpose of each of the *f*_*i*_ functions is shown in Table 1, and they have been determined from work that defines some of the key parameters of glucose and insulin metabolism in function form. References to the original physiological experiments may be found in [34]. Equation (2.1) were solved as described previously [41].

**Table 1:** Descriptions of the functions *f*_1_ through *f*_7_, *G*_*in*_(*t*) and *I*_*in*_(*t*)

Function name	Description
*f*_1_(*G*(*t*−*τ*_1_))	Insulin production and release by pancreas
*f*_2_(*G*(*t*))	Insulin-independent glucose utilization
*f*_3_(*G*(*t*))×*f*_4_(*I*(*t*))	Insulin-dependent glucose utilization
*f*_5_(*I*(*t*−*τ*_2_))	Hepatic glucose production
*f*_6_(*G*(*t*))	Inhibitor of hyperglycemia
*f*_7_(*G*(*t*)−330)	Inhibitor of hyperglycemia
*G*_*in*_(*t*)	Nutritional source
*I*_*in*_(*t*)	Exogenous insulin

### Model parameters and what they represent

The model incorporates 6 adjustable parameters: *τ*_1_,*τ*_2_,*d*_*i*_,*α*,*β*, and г, which have been boldfaced in Eq. (2.2) for easy identification; their value determines the specific pathophysiology of the patient being simulated. Each parameter affects only a portion of the glucose-insulin axis; this principle is summarized in Table 2. A detailed description of each model parameter follows next.

**Table 2:** A summary of the 6 parameters of the model and their purpose

Parameter name	Description
*τ*_1_	Time delay, endogenous insulin secretion
*τ*_2_	Time delay, endogenous glucose secretion
*β*	Amplitude, insulin-dependent glucose utilization
*α*	Amplitude, endogenous insulin production
г	Amplitude, gluconeogenesis
*d*_*i*_	Amplitude, insulin clearance

The model was validated for normal physiology and diabetes (that is, for normal rates of gluconeogenesis or г=1 in Eq. (2.2a)) by showing that detailed glucose measurements from several healthy subjects and patients with type 1 or type 2 diabetes can be reproduced when the parameter values are appropriately chosen [38]. The model correctly captures the effects of insulin therapy and a nutritional source. The reverse process was also found to be valid because an unknown patient’s corresponding parameter values can be used to correctly diagnose the patient’s underlying pathophysiology [38].

Type 1 diabetes was mainly captured by decreasing the term that describes insulin secretion by a constant, *α*, in Eq. (2.2c). Similarly, type 2 diabetes was primarily modeled by decreasing the term that describes insulin-dependent glucose utilization by another constant, *β*, in Eq. (2.2b). When *α* and *β* are appropriately chosen and less than one, the virtual patient will either secrete a suboptimal amount of insulin or demonstrate *IR*.

SH in critical illness is the result of at least 4 factors: increased gluconeogenesis [1–3], increased *IR* [4–9], *ID* from decreased pancreatic insulin secretion (*ID*_*p*_) [10], and *ID* from increased insulin clearance (*IC*) [5, 10]. Each factor was incorporated into our model. For example, an increase in the hepatic glucose production term by a constant г (where г is greater than unity, Eq. (2.2a)) increases gluconeogenesis [41]. It was assumed that the shape of *f*_5_ (the function that represents gluconeogenesis) does not change with elevated rates of gluconeogenesis because it has been shown that the shapes of the *f*_*i*_ functions are more important than their exact functional form in reproducing the correct glucose-insulin dynamics [43]: a reasonable assumption for a qualitative study.

Insulin resistance in SH was modeled by decreasing the rate of insulin-dependent glucose utilization, as with type 2 diabetes by setting *β*<1. Reduced insulin secretion in SH was also simulated in the same fashion as with type 1 diabetes by fixing *α*<1. Finally, critical illness has been shown to increase *IC* by 50–70 % in human studies [5, 10]. Experiments have demonstrated that the process of insulin degradation is proportional to its concentration, and this proportionality constant is *d*_*i*_ in Eq. (2.2d) [44]. Thus, increasing *d*_*i*_ by 50 % increases the *IC*, which we defined as *IC*_50_.

It is the time delays that allow the model to reproduce the naturally occurring glucose-insulin ultradian oscillations [35, 36]. They describe the time required for the body to produce and secrete insulin, *τ*_1_ in *f*_1_(*G*(*t*−*τ*_1_)), as well as to produce and secrete glucose, *τ*_2_ in *f*_5_(*I*(*t*−*τ*_2_)).

### How the model parameter values were chosen

The amount of *IR*, *ID*_*p*_ and increased *IC* that occurs in SH varies among patients and is not well defined. To proceed, we have slightly modified the validated *α*,*β* and *d*_*i*_ values that were found to represent normal physiology (see *sim*1 in [38]) to define low normal pancreatic insulin secretion (mild *ID*_*p*_), mild *IR* and high normal IC, respectively. Next, we decreased the modified *α* and *β* values by 25 *%* to represent severe *IR* and severe *ID*_*p*_. This defined the pancreatic dysfunction and the decreased insulin-dependent glucose utilization severity in our virtual patients. For reference, the validated *α* and *β* values required to accurately model type 1 and type 2 diabetes are an additional 20 *%* smaller than the parameter values used to represent severe *ID*_*p*_ and severe *IR* [38]. Thus, we assumed that the level of pathology being simulated was reasonable in that the glucose dynamics produced wound not be abnormally divergent from clinically familiar pathology. Finally, we used the experimentally determined observation on humans that *IC* may be increased by 50–70 *%* in critical illness [10] to justify increasing the high normal *d*_*i*_ value by 50 % (called *IC*_50_). A summary of the model parameters with their associated descriptors is found in Table 3. We have limited the data analysis to include only qualitative trends in the measurable quantities: the change-in-*MGC* and the change-in-*GV* versus the dose of SQ insulin injected, as well as hypoglycemic episodes.

**Table 3:** Summary of model parameter values, description of the resultant pathophysiology, with associated descriptor

Name and value	Pathophysiology or function	Descriptor
*β*=0.8	Low normal insulin-dependent	Mild *IR*
	glucose utilization	
*β*=0.6	Low normal insulin-dependent	Severe *IR*
	glucose utilization decreased by 25 %	
*α*=0.8	Low normal insulin secretion	Mild *I* *D*_*p*_
*α*=0.6	Low normal insulin secretion	Severe *I* *D*_*p*_
	decreased by 25 %	
*d*_*i*_=0.17	High normal *IC*	Borderline *IC*
*d*_*i*_=0.25	High normal *IC* increased by 50 %	*I* *C*_50_
$\digamma = 2$	Normal gluconeogenesis rate doubled	No descriptor

For the time delays, normal values were used [38]; however, they were chosen so that all of the 8 virtual patients simulated had a maximum glucose value between 150 and 170 mg/dL [41]. This approach facilitated interpatient comparison of the change-in-*MGC* and change-in-*GV*.

### Modeling SQ insulin

The durations of action of SQ Lispro insulin and SQ regular insulin were taken to be 240 and 480 min, respectively; their onset of action was defined to be 5 and 30 min, respectively [31]. For each virtual patient, 25 injections of SQ Lispro insulin and SQ regular insulin were given with doses that ranged from 0 to 6 U. The upper insulin limit of 6 U was chosen because this is near the dose where insulin’s effects begin to saturate for a 70-kg body mass [45].

SQ insulin injections, *I*_*in*_(*t*) in Eq. (2.2c), were modeled with simple, linear piecewise functions [33, 41]. For example, 5 min after a SQ injection of Lispro insulin, the absorption was assumed to linearly increase to a maximum value during the first 30 min, followed by a linear decrease to nearly zero by minute 120; this was followed by a small residual tail for the remaining 120 mins. The main form of this function is a triangle with an area that represents the total number of SQ Lispro insulin units injected. The explicit functional forms that represent the SQ insulin injections are shown next.

The SQ Lispro insulin injections were modeled with [33, 41]:

(2.3) $$ I_{{\text{Lis}}}(t) = \left\{ \begin{array}{rcl} 0.25 & \text{for}& 0 \leq t \leq 5, \\ 0.25 + \left(1+ {{t-30}\over{30-5}}\right) & \text{for} & 5 \leq t < 30, \\ 0.25 + \left(1- {{t-30}\over{120-30}}\right) & \text{for} & 30 \leq t < 120, \\ 0.25 & \text{for}& 120 \leq t \leq 240. \end{array}\right.  $$

Similarly, for the SQ regular insulin injections, the following function was used [33, 41]:

(2.4) $$ I_{{\text{Reg}}}(t) = \left\{ \begin{array}{rcl} 0.25 & \text{for} & 0 \leq t \leq 30, \\ 0.25 + \left(1+ {{t-120}\over{90}}\right) & \text{for} & 30 \leq t < 120, \\ 0.25 + \left(1- 0.5 \times {{t-120}\over{120}}\right) & \text{for} & 120 \leq t < 240, \\ 0.25 + 0.5 \times \left(1- {{t-240}\over{240}}\right) & \text{for} & 240 \leq t \leq 480. \end{array}\right.  $$

In both functions, *t* represents time; the time of injection is *t*=0.

### Measuring the change-in-GV and change-in-MGC

The change-in-*MGC* and change-in-*GV* are the metrics of interest, and they were calculated as follows: *MGC*/*GV* was measured after an SQ insulin injection from which the baseline *MGC*/*GV* was subtracted. We have adopted the term “baseline” to refer to data that were gathered from the virtual patient when 0 U of insulin was injected. Thus, the patients (having received no insulin) served as their own controls.

*MGC* was calculated as the sum of glucose concentrations that occur each second after the beginning of the simulations (*t*=0) divided by the total number of glucose concentrations used in the sum; the time over which the calculation is performed was 750 mins (the duration of each simulation).

*GV* does not have a standard definition [46]; this reflects the lack of solid knowledge on what exactly in abnormal glucose-insulin dynamics causes an increase in adverse outcomes that has been associated with increased *GV* in literature. In several retrospective studies, different indices have been proposed; for example, glucose variability index [47], standard deviation [15, 48], glycemic lability index [49] and mean amplitude of glucose excursions [50]. It has to be mentioned that blood glucose values are seldom normally distributed, a mathematical condition for use of standard deviation [51]. In literature, this limitation is mostly ignored. In a systemic review of *GV*, and its effect on mortality, each study reviewed found at least one measure of *GV* that was associated with mortality [52].

A definition of *GV* well suited for this study is the mean absolute glucose change [53, 54]. This is the simple summation of all absolute changes in glucose concentrations, divided by the time over which measurements were taken. In this way, two excursions of identical extent, but differing in duration, contribute differently to the overall sum of variability. The downside of such a measure in clinical practice is that noise, a particular problem in continuous glucose concentration monitoring, is hard to separate from the signal [55]. Concerning the data gathered from computer models of glucose-insulin dynamics two observations are relevant: (1) there is no noise within the data generated by simulation and (2) the time over which glucose measurements are made can be constructed to be identical. In such a scenario, time may be suppressed in the mean absolute glucose change calculation, as it is the same for each *GV* measurement.

We thus have chosen to define *GV* as the average distance between adjacent local maximum and local minimum values in a patient’s *G*(*t*) data (i.e. mean absolute glucose change); the time over which the calculation is performed was again 750 mins. Because all *GV* measurements occurred over exactly the same relatively short time interval, the change-in-*GVs* were not normalized by the time over which the data was gathered. Finally, the local maximum/minimum values in the *G*(*t*) data were designated as peaks/troughs.

### Modeling continuous nutrition

The 3 rates of continuous nutrition, *G*_*in*_(*t*) in Eq. (2.2a), were 135 mg/min (nutrition at goal), 67.5 mg/min (nutrition at half goal), and 0 mg/min (no nutrition); see Table 4 for a summary. For example, *G*(*t*) = 135 mg/min is equivalent to 82.2 mL/h of Glucerna®;1.2 Cal, which would provide 2367 calories and 194 g of net carbohydrates in 24 h.

**Table 4:** A summary of the different rates of continuous nutrition studied with an associated descriptor

Name and value	Descriptor
*G*_*in*_(*t*)=135 mg/min	Nutrition at goal
*G*_*in*_(*t*)=67.5 mg/min	Nutrition at half goal
*G*_*in*_(*t*)=0 mg/min	No nutrition

### Explicit forms of the glucose-insulin metabolic functions

For completeness, the explicit forms of the *f*_*i*_ functions are presented:

(2.5a) $$\begin{array}{*{20}l}  & f_{1}(G(t-{\tau}_{1})) = \ {{R_{c}}\over{1+ exp((c_{1}-(G(t-{\tau}_{1})/V_{g}))(1/e_{1}))}}, \end{array} $$

(2.5b) $$\begin{array}{*{20}l}  & f_{2}(G(t)) = \ U_{b} \times [1-exp(-(G(t)/c_{2}V_{g})], \end{array} $$

(2.5c) $$\begin{array}{*{20}l}  & f_{3}(G(t)) = \ {{G(t)} \over {c_{3} V_{g}}}, \end{array} $$

(2.5d) $$\begin{array}{*{20}l}  & f_{4}(I(t)) = \ U_{o} + {{U_{c}-U_{o}}\over{1+exp(-\kappa log((I(t)/c_{4})(1/V_{c})+(1/{Et}_{c})))}}, \end{array} $$

(2.5e) $$\begin{array}{*{20}l}  & f_{5}(I(t-{\tau}_{2})) = \ {{ R_{g}}\over{1+exp(e_{1}((I(t-{\tau}_{2})/V_{p})-c_{5}))}}, \end{array} $$

(2.5f) $$\begin{array}{*{20}l}  & f_{6}(G(t)) = \ {{1}\over{exp(\gamma ((G(t)/c_{3} V_{g})- c_{6}))}}, \end{array} $$

(2.5g) $$\begin{array}{*{20}l}  & f_{7}(G(t)-330) = \ S_{b} + {{S_{c}-S_{b}}\over{1+exp(\delta(((G(t)-330)/c_{3}V_{g})-c_{7}))}}. \end{array} $$

The associated constant values within each function are derived from human physiological data [34] and have been presented in tabulated form [38, 41].

## Results

The results are grouped in subsections by insulin therapy, patient pathophysiology, and the rate of the nutritional source. In general, graphs depicting the change-in-*MGC* show a decrease in *MGC* when a data point is below the *y*=0 line and an increase when the data point is above *y*=0. The same is true for the change-in-*GV* graphs. Note that all insulin injections were timed to be given at a glucose concentration peak.

### SQ Lispro insulin, the 4 borderline *IC* patients, and nutrition at goal

This subsection contains the change-in-*GV* and change-in-*MGC* data for the 4 patients with borderline *IC*. All patients received nutrition at goal, and SQ Lispro insulin was used to treat their hyperglycemia.

**Fig. 1:**
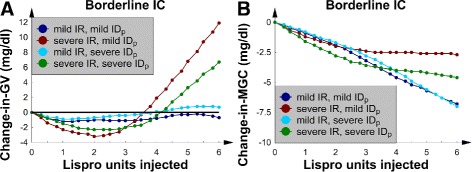
The 4 patients with borderline *IC* and nutrition at goal. **a** Change-in-*GV* versus number of SQ Lispro insulin units injected. **b** The change-in-*MGC* versus number of SQ Lispro insulin units injected

SQ Lispro insulin injections were noted to lower *GV* when the insulin dose was less than 4 U (Fig. 1a). In the 2 patients with mild *IR*, *GV* remained relatively unaffected by the dose because the change-in-*GV* curves were nearly flat and near *y*=0. For the 2 patients with severe *IR*, however, the *GV* increased when the doses were greater than 4 U. Insulin resistance also affected the shape of the change-in-*MGC* curves (Fig. 1b). Patients with mild *IR* had a change-in-*MGC* curve that decreased linearly as the SQ Lispro insulin dose increased, which was an intuitive result. Nevertheless, patients with severe *IR* had *MGC*s that were not significantly decreased as the dose of SQ Lispro insulin was increased beyond 4 U. This result implies that *IR* significantly affected the response of the glucose-insulin axis to an SQ ultrashort insulin analog. In comparison, changing *ID*_*p*_ from mild to severe did not significantly alter the effect of SQ Lispro insulin on either *MGC* or *GV*.

**Fig. 2:**
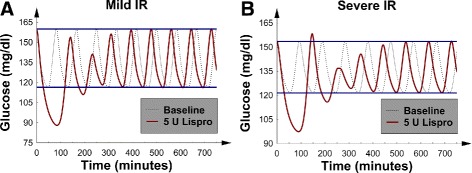
*G*(*t*), glucose concentration versus time, after 5 U of SQ Lispro insulin were injected at *t*=0; baseline *G*(*t*) was also plotted for comparison. Both patients were receiving nutrition at goal. **a** The patient with mild *IR*, mild *I* *D*_*p*_ and borderline *IC*. **b** The patient with severe *IR*, mild *I* *D*_*p*_ and borderline *IC*; note the “rebound hyperglycemia”

To interpret the change-in-*MGC* and change-in-*GV* graphs in terms of the underlying glucose concentration dynamics, the detailed *G*(*t*) data were examined for 2 selected patients: 1 with mild *IR* and another with severe *IR*. Both patients had mild *ID*_*p*_, and 5 U of SQ Lispro insulin was injected at a glucose concentration peak at time *t*=0. For syntax, the first peak and first trough occurred just after the insulin injection, and this convention is used below. The corresponding baseline *G*(*t*) data (no insulin injected) were also plotted for comparison. For the patient with mild *IR* (Fig. 2a), the first *G*(*t*) trough was significantly less than the baseline trough. Additionally, the first and second peaks were significantly decreased. These factors combined to lower both *GV* and *MGC*.

The more interesting behavior was exhibited by the patient with severe *IR* (Fig. 2b). The first glucose concentration peak was seen to be larger than its corresponding pre-insulin baseline peak, and we termed this behavior “rebound hyperglycemia.” Furthermore, the first glucose concentration trough after the injection was significantly lower than the corresponding baseline trough. These 2 factors combined to decrease *MGC* but increase *GV*. The “rebound hyperglycemia” first appeared at 4 U and worsened as the dose of SQ Lispro insulin was increased. Because of the “rebound hyperglycemia”, the change-in-*MGC* curves (Fig. 1b) began to flatten when the *IR* was severe and the dose of SQ increased above 4 U. This picture signaled a nonlinear dose-response relation between SQ Lispro insulin and *MGC* in patients with severe *IR*: a potential important observation for the design of sliding-scale SQ Lispro insulin protocols for such patients. No episodes of hypoglycemia were noted; however, the significantly decreased first trough in Fig. 2b suggests that SQ Lispro insulin may be particularly likely to cause hypoglycemia. It is important to mention that “rebound hyperglycemia” was also seen when type 2 diabetics were modeled with a validated numerical model that simulated diabetics receiving continuous nutrition while being treated with SQ Lispro insulin for their hyperglycemia [42].

In general, *IR* seems to have rendered the glucose-insulin axis more sensitive to perturbations caused by SQ ultrashort insulin analogs; this result was evidenced by the “rebound hyperglycemia” and increased *GV*. It is important to note that hourly glucose checks would be required to identify an increase in *GV* in an analogous real patient.

### SQ regular insulin, the 4 borderline *IC* patients, and nutrition at goal

The same group of 4 patients from the previous subsection (the 4 patients with borderline *IC* who were receiving nutrition at goal) were next given SQ regular insulin at exactly the same time point as the previous SQ Lispro insulin injections; this approach facilitated a comparison between the 2 insulin preparations.

**Fig. 3:**
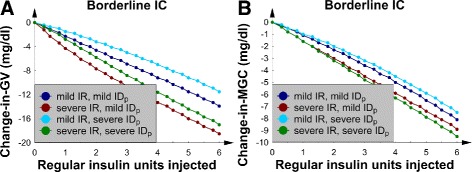
The 4 patients with borderline *IC* and nutrition at goal. **a** The change-in-*GV* versus number of SQ regular insulin units injected. **b** The change-in-*MGC* versus number of SQ regular insulin units injected

**Fig. 4:**
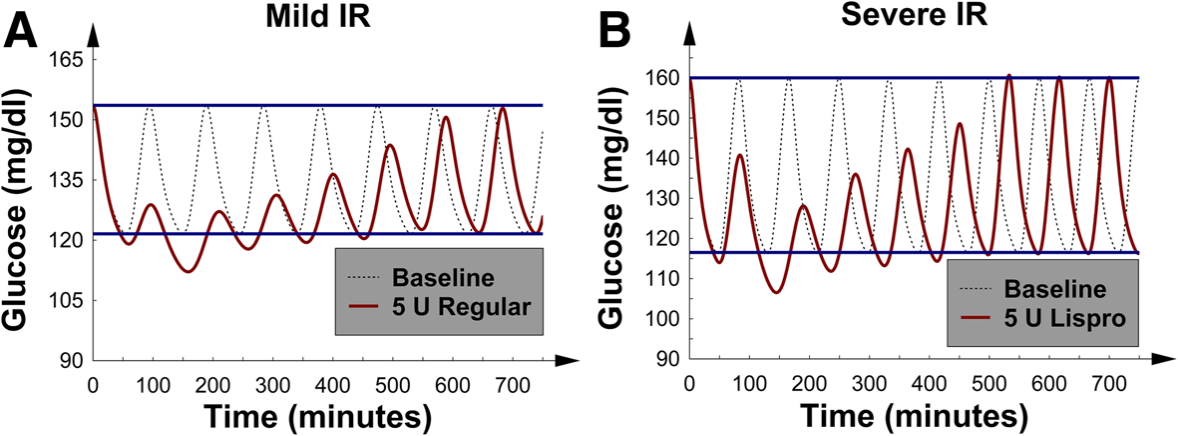
*G*(*t*), glucose concentration versus time, after 5 U of regular insulin were injected at *t*=0; baseline *G*(*t*) was also plotted for comparison. Both patients were receiving nutrition at goal and had mild *I* *D*_*p*_ and borderline *IC*. **a** The patient with mild *IR*. **b** The patient with severe *IR*

Figure 3a and b show linear decreases in *GV* and *MGC* with an increasing SQ regular insulin dose. This picture demonstrated a linear dose-response relation between SQ regular insulin and *MGC*/*GV*; a linear dose-response is presumed optimal for a sliding-scale SQ insulin protocol. To interpret these metrics in terms of the glucose concentration dynamics, the *G*(*t*) data were again examined; Fig. 4a shows a patient with mild *IR* and mild *ID*_*p*_. The results revealed that the first 5 glucose concentration peaks were lowered; this finding is consistent with the duration of action of SQ regular insulin.

In contrast to SQ Lispro insulin, the same well-behaved dynamics occurred in the patient with severe *IR*; again, the first several glucose concentration peaks were suppressed (Fig. 4b). Furthermore, SQ regular insulin only minimally decreased the glucose concentrations troughs as compared to SQ Lispro insulin (Figs. 2 and 4). This finding suggests that SQ regular insulin would be less likely to cause hypoglycemia, as compared to SQ Lispro insulin (at least, for the patients examined thus far). Finally, both *MGC* and *GV* were similarly lowered regardless of the *IR* or *ID*_*p*_ value. Unlike SQ Lispro insulin, SQ regular insulin did not cause “rebound hyperglycemia” at higher doses. In addition, no episodes of hypoglycemia were noted.

In this subsection, the response of the glucose-insulin axis to perturbation by a short SQ insulin preparation appeared better behaved as compared to the effects of the ultrashort SQ Lispro insulin (see the previous subsection). Thus, the difference between the onset/duration of action of the insulin preparations significantly affects the response of the glucose-insulin axis to SQ insulin therapies as measured by *GV* and *MGC*. Similar results were observed in a validated numerical study of patients with type 2 diabetes who received continuous enteral feeding [42].

### SQ Lispro insulin, the 4 patients with *IC*_50_, and nutrition at goal

To examine the consequences of increasing *IC* to pathological levels, the second set of 4 patients with *IC*_50_ (borderline *IC* increased by 50 %) was studied next; this analysis allowed for a comparison of the glucose dynamics in the patients with borderline *IC* and *IC*_50_ after SQ Lispro insulin therapy. As before, nutrition was at goal.

**Fig. 5:**
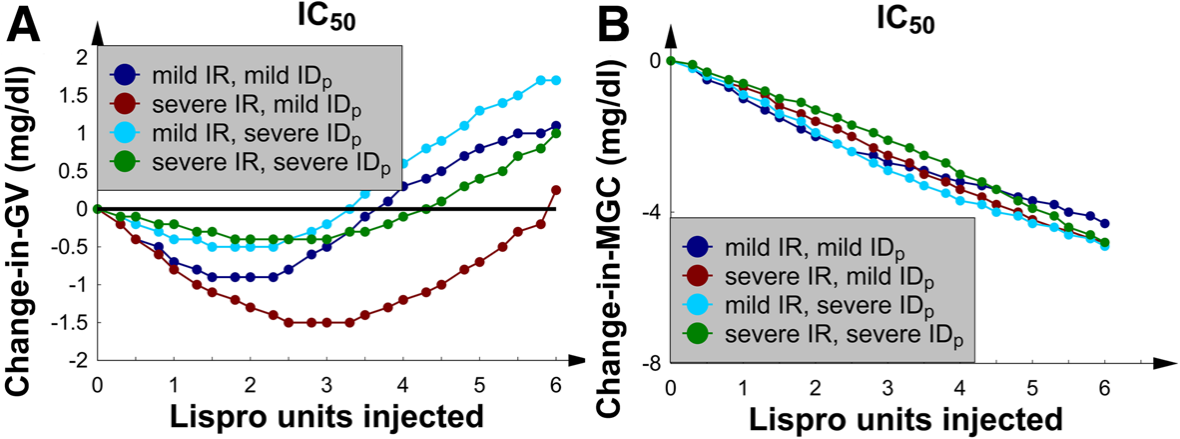
The 4 patients with *I* *C*_50_ (50 % increase in IC from borderline *IC*) and nutrition at goal. **a** The change-in-*GV* versus number of SQ Lispro insulin units injected. **b** The change-in-*MGC* versus number of SQ Lispro insulin units injected

A direct comparison of the borderline *IC* and *IC*_50_ change-in-*GV* curves (Figs. 1a and 5a) shows that the glucose dynamics remained qualitatively similar between the 2 patient groups. In both sets of curves, *GV* first decreased and then increased after approximately 4 U. In contrast, the change-in-*GV* was an order of magnitude smaller in the *IC*_50_ patient group. This result was due to the additional mechanism (an increased *IC*) that worsened *ID* and blunted the patient’s response to the same SQ Lispro insulin dose. The *IC*_50_ change-in-*MGC* curves (Fig. 5b) were different from the corresponding change-in-*MGC* graphs with borderline *IC* (Fig. 1b) because the *MGC* decreased in a monotonic fashion and never flattened.

**Fig. 6:**
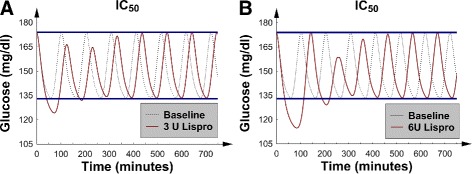
*G*(*t*), glucose concentration versus time. Baseline *G*(*t*) was also plotted for comparison. The patient had severe *IR*, mild *I* *D*_*p*_ and *I* *C*_50_ with nutrition at goal. *G*(*t*) after **a** 3 U of SQ Lispro insulin were injected at *t*=0 and after **b** 6 U of SQ Lispro insulin were injected at *t*=0

To demonstrate that the qualitative behavior of the measurables remained similar between the borderline *IC* and *IC*_50_ patient groups, the effects of increasing the SQ Lispro insulin dose on a patient with severe *IR* were examined. In particular, Fig. 6a and b show the effects of 3 and 6 U of SQ Lispro insulin, respectively, which were injected in the patient with severe *IR* and mild *ID*_*p*_. The graphs show that as the dose of SQ Lispro insulin increased, the *G*(*t*) curves began to resemble the curves that were found in the patient with severe *IR*, mild *ID*_*p*_, and borderline *IC* (Fig. 2b). That is, the first peak after the SQ Lispro insulin injection nearly returned to its baseline value, just avoiding “rebound hyperglycemia”; note, this phenomenon occurred at the higher insulin doses. Therefore, the underlying glucose-insulin dynamics remained qualitatively similar between the borderline *IC* and *IC*_50_ patient groups; albeit, the effects of SQ Lispro insulin were blunted by the higher *IC* values. In fact, “rebound hyperglycemia” could be produced when the SQ Lispro insulin dose exceeded 6 U (data not shown). Finally, changing *ID*_*p*_ from mild to severe did not significantly affect the change-in-*MGC* and change-in-*GV* curves; this finding is similar to the data in the corresponding borderline *IC* subsection.

### SQ regular insulin, the 4 patients with *IC*_50_, and nutrition at goal

**Fig. 7:**
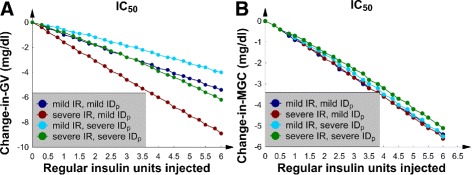
The 4 patients with *I* *C*_50_ and nutrition at goal. **a** The change-in-*GV* versus number of SQ regular insulin units injected. **b** The change-in-*MGC* versus number of SQ regular insulin units injected

Next, the previous 4 numerical experiments were repeated; however, the insulin preparation was changed to regular insulin. In the 4 patients with *IC*_50_, Fig. 7a and b show that both *GV* and *MGC* decreased in a linear manner as the SQ regular insulin dose was increased. The corresponding *G*(*t*) graphs were similar to those patients with borderline *IC* presented in Fig. 4; therefore, the *G*(*t*) data for the *IC*_50_ patients are not shown. Moreover, the linear dose-response relation between SQ regular insulin and *MGC* was found to be similar to the dependence shown in Fig. 3b: again, presumed optimal for a sliding-scale SQ insulin protocol. Now familiar, changing *ID*_*p*_ from mild to severe did not significantly affect the change-in-*MGC* and change-in-*GV* curves. Furthermore, we noted that increasing the *IC* from borderline to *IC*_50_ resulted in approximately a 50 % decrease in the size of the change-in-*GV* and change-in-*MGC* curves (compare Figs. 3 and 7).

### Both SQ insulin preparations, all 8 patients, and nutrition at half goal

It is not uncommon for patients to require a decrease in the rate of their enteral feedings because of intolerance; therefore, all of the above experiments were repeated but with the rate of nutrition halved, or nutrition at half goal. All 8 patients are discussed within this subsection.

**Fig. 8:**
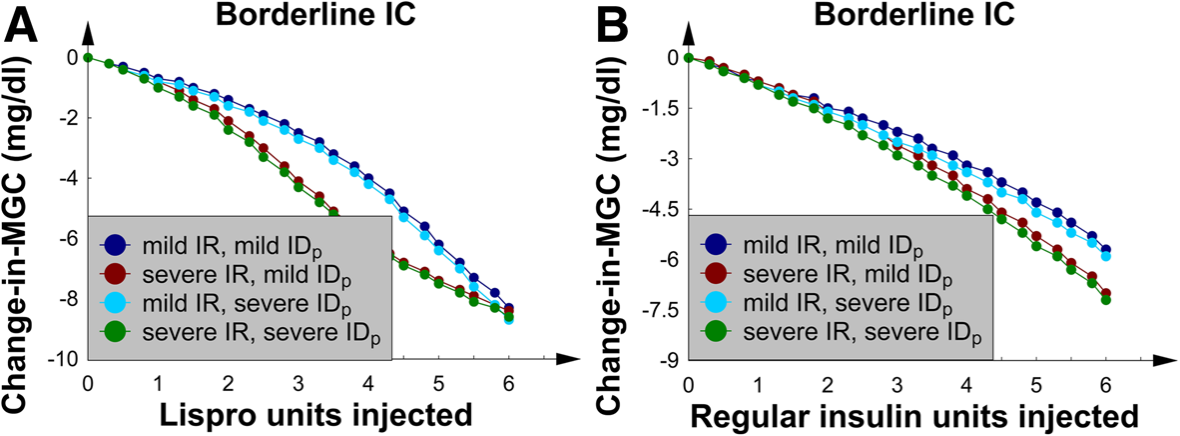
The 4 patients with borderline *IC* and nutrition at half goal. **a** The change-in-*MGC* versus number of SQ Lispro insulin units injected. **b** The change-in-*MGC* versus number of SQ regular insulin units injected

**Fig. 9:**
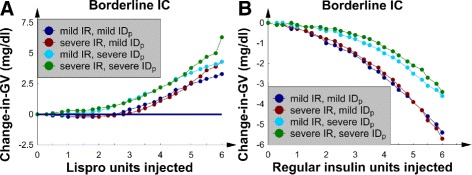
The 4 patients with borderline *IC* and nutrition at half goal. **a** The change-in-*GV* versus number of SQ Lispro insulin units injected. **b** The change-in-*GV* versus number of SQ regular insulin units injected

For the 4 patients with borderline *IC*, both insulin preparations decreased *MGC* in a linear manner (Fig. 8a and b). Nevertheless, SQ Lispro insulin and SQ regular insulin had an opposite effect on *GV*. SQ Lispro insulin increased *GV* as the dose increased (Fig. 9a), whereas SQ regular insulin decreased *GV* (Fig. 9b). The *G*(*t*) data showed that the main effect of SQ Lispro insulin was to significantly decrease the first glucose concentration trough after the injection, suggesting that SQ Lispro insulin has the larger risk of causing hypoglycemia when the rate of continuous nutrition is decreased, when compared to SQ regular insulin. After this, the first postinjection glucose concentration peak returned to its baseline value, nearly causing “rebound hyperglycemia”. This phenomenon decreased *MGC* but increased *GV*. The SQ regular insulin injection, however, smoothly decreased several glucose concentration peaks, thus decreasing both *MGC* and *GV*, just as in Fig. 3; therefore, the *G*(*t*) data are not shown.

Increasing the *IC* from borderline to *IC*_50_ decreased the magnitude of the change-in-*MGC* and change-in-*GV* curves; this pattern was seen in the corresponding subsections when the nutrition was at goal. Because the qualitative shapes of the curves were similar to those in the previous subsections, the data are not shown.

Finally, there were no episodes of either hypoglycemia or “rebound hyperglycemia” in any patient, and the *ID*_*p*_ value again did not significantly affect the change-in-*MGC* and change-in-*GV* curves.

### Both SQ insulin preparations, all 8 patients, and no nutrition

Either because of a required procedure or feeding intolerance, patients sometimes need their enteral feedings held. To examine this clinical scenario, the nutritional source was removed, and the simulations were repeated; all 8 patients are discussed within this subsection.

The main effect of removing the nutritional source on the baseline simulations was a decrease in the glucose concentration troughs that was greater than the decrease in the corresponding glucose concentration peaks. In the comparison with the nutrition-at-goal simulations, the baseline glucose concentration troughs were approximately 20 mg/dL lower. The change-in-*GV* and change-in-*MGC* curves were quantitatively similar to the corresponding curves in the nutrition-at-half-goal subsection (data not shown).

The most remarkable finding in this subsection was that SQ Lispro insulin (but not regular insulin) caused hypoglycemia (glucose concentrations near 60 mg/dL). This effect was seen in the 4 patients with borderline *IC* when the dose exceeded 4 U; the effect occurred in part because the baseline troughs were lowered when the nutritional source was removed.

Finally, increasing the *IC* from borderline to *IC*_50_ decreased the magnitude of the change-in-*GV* and change-in-*MGC* curves. The *ID*_*p*_ value again had minimal effects on the glucose dynamics.

## Discussion

To the best of our knowledge, SQ Lispro insulin and SQ regular insulin have not been compared in SH patients receiving continuous nutrition. The numerical study that included this comparison, unfortunately, did not involve analysis of *ID* [41], which we have included here. Our 3 main findings are:

1. SQ Lispro insulin tended to increase *GV* even if *MGC* was decreased, and at times, it did so in a nonlinear manner. Hourly glucose checks would be required to uncover this phenomenon in a corresponding real patient.
2. SQ regular insulin tended to decrease *MGC* and *GV* in a linear manner, with respect to its dose, suggesting that this preparation is better suited for a sliding-scale SQ protocol, as compared to SQ Lispro insulin.
3. Higher SQ Lispro insulin doses produced hypoglycemia when the patients’ nutritional sources were discontinued; SQ regular insulin did not cause hypoglycemia in any simulation.

An additional finding of interest is that severe *IR* seems to predict when the glucose-insulin axis has increased sensitivity to changes in endogenous insulin concentrations. It was found that such increased sensitivity could result in “rebound hyperglycemia” and increased *GV* when the continuous nutrition was at goal, provided an ultrashort SQ insulin analog was used.

### Glucose variability

Because it is associated with negative clinical outcomes, *GV* was chosen as a primary end point [14, 15, 21, 48, 49, 52, 53, 56, 57]. Glucose fluctuations produce changes in plasma osmolality that can lead to cellular and organ dysfunction, thus increasing morbidity [58]. Oxidative stress, which is enhanced by glucose fluctuations more than by sustained hyperglycemia [59], may be a unifying mechanism underpinning vasoconstriction, microvascular thrombosis, and inflammation associated with elevated *GV* [60]. One study of surgical critical-care patients showed that the highest mortality rate is observed when *GV* and hyperglycemia were both present; that study also revealed that *GV* is the more important outcome predictor [53]. Such studies have led some to argue that glucose management protocols should focus on both *MGC* and *GV* as treatment targets [61].

There seem to be no clinical studies that involve measurement of *GV* after administration of short-acting SQ insulin preparations within the time frame examined here. This is probably because sliding-scale SQ insulin regimens imply sampling of glucose values every 3-6 h. The data presented in Figs. 2, 4 and 6 show that one glucose measurement per hour would be required to detect the increase in *GV* uncovered by our numerical simulations. Therefore, the ability of a typical sliding-scale insulin protocol to capture the effects of SQ Lispro insulin on *GV* is limited.

### Effects of insulin resistance

The *GV* data suggested that patients with severe *IR* are the most sensitive to changes in exogenous insulin concentrations. “Rebound hyperglycemia” occurred only in the patients with severe *IR* when the continuous nutrition was at goal. This result suggests that these patients are at a higher risk of developing elevated *GV* from an insulin therapy, particularly if the insulin concentration changes with sufficient amplitude. This notion is consistent with the numerical works that examined the effects of insulin infusion rate changes on patients with SH [62] and on patients with type 2 diabetes who were receiving continuous enteral feedings and SQ insulin therapy [42]. It should be noted, that the model used to examine the diabetics is the one used in this study, albeit with $\digamma =1$ (normal rate of gluconeogenesis); furthermore, the model parameters used were validated against actual patients’ glucose concentration data. Currently, there is no easy method of directly measuring the *IR* and what constitutes severely elevated *IR* is poorly defined. Computer-based insulin protocols that individually construct patient centered insulin therapies can provide the *IR* data [63], which may indicate impending infection if the *IR* is elevated in patients without diabetes [64, 65].

We hypothesize that *IR* is an important metric for the categorization of SH disease states because our virtual patients’ glucose dynamics varied significantly according to its value. Perhaps severe *IR*, and its associated altered glucose mechanics, may partially explain why the treatment of SH in the TGC era correlated negatively with GV (as measured by standard deviation) [15]. Furthermore, patients with various relative values of *IR* might behave differently enough (under non-patient-centered insulin therapy) to explain, in part, why certain subgroups, such as trauma patients and patients receiving steroids, seem to benefit from TGC while other subgroups do not [22, 23]. On the other hand, the direct *IR* data (paired with the etiology of critical illness) does not exist to support this query. It is noteworthy, however, that the SPRINT study, a computer-based insulin protocol for the critically ill that determines and incorporates each patient’s *IR*, showed no significant association between glucose variability or mortality in either the SPRINT or TGC arm [66].

### Effects of insulin deficiency

Unlike *IR*, worsening *ID* did not correlate with increased *GV*. Severe *ID*, which was a result of decreased pancreatic insulin secretion and an elevated *IC*, was associated with smaller decreases in *MGC* and *GV* after both insulin therapies, as compared to patients with borderline *ID*.

### Importance of time scales

We posit that *GV* may be increased by an insulin therapy when the time scale over which the insulin concentration changes is of the same order, or smaller, than the period of the patient’s glucose ultradian oscillation. A similar mechanism (uncovered by a validated numerical study) was speculated to be present in type 2 diabetics who were receiving continuous enteral feedings and SQ insulin therapies for their hyperglycemia [42].

We arrived at this hypothesis after comparing two length scales that were present in each simulation: the period of the patient’s ultradian glucose concentration oscillations and the duration of action of the insulin preparation. The period of the ultradian oscillations in glucose represented the time needed by the glucose-insulin feedback system to maintain glucose concentrations within a particular range and variability. When the change in exogenous insulin concentration within this time frame was large, the glucose concentrations fluctuated, and *GV* increased. In particular, the SQ Lispro insulin absorption/concentration profile increased in the first 35 min after the injection and then decreased to nearly zero over the next 90 min, nearly matching the period of the patients’ ultradian glucose concentration oscillations. In comparison, SQ regular insulin reaches its maximum after nearly 1 ultradian period, and almost 2 ultradian periods are required for its concentration to reach zero [33, 41].

### Effects of the rate of nutrition

The rate of the continuous nutritional source also affected the patient’s response to the SQ insulin therapies. For the doses examined, SQ regular insulin lowered *MGC* and *GV* without causing hypoglycemia. SQ Lispro insulin caused hypoglycemia only at higher doses if the nutritional source was discontinued. This result is consistent with recent clinical [67] and numerical [42] studies of patients with diabetes who received continuous gastric feeds. It is also important to note that the rate of continuous nutrition also played a role in whether SQ Lispro insulin produced “rebound hyperglycemia”.

These results highlight the importance of considering a patient’s nutritional intake when designing an insulin treatment protocol [68]. The most accurate insulin protocol would be patient centered and responsive to the patient’s unique physiology and nutritional state. In fact, some computerized protocols also make recommendations as to the nutritional rates the patients should be receiving so that hypoglycemia, and perhaps increased *GV*, may be avoided if the insulin infusion rate is changed [69]. This make sense, as the dynamics of glucose and insulin arise from a set of coupled systems (Eq. (2.1)). There are several computational tools that have been examined in the critical care [66, 69–71] and outpatient settings [72, 73]. The field has a rich history surveyed in reviews [74–76].

### Limitations

This study has several limitations. First, although the model was validated for type 1 and type 2 diabetes, it had not been validated on human data gathered from the critically ill. As such, we have discussed only the qualitative aspects of our data. This study should be followed with a randomized controlled clinical study that directly compares SQ Lispro insulin and SQ regular insulin in patients with SH receiving continuous nutrition before a clinical recommendation can be made. *GV* should remain a primary end point, which would require continuous glucose monitoring or glucose checks every hour for evaluation [77]. Finally, this study did not address insulin stacking, which may also cause hypoglycemia [78].

## Conclusion

SH and different SQ insulin therapies can be studied using a mathematical model of the glucose-insulin feedback system. This type of study allows for qualitative analysis of *MGC*, *GV*, and hypoglycemia after SQ injections of Lispro and regular insulins. The model yields insights into the dynamics of glucose metabolism that would be difficult to ascertain otherwise. The model may also guide the design of future clinical trials in, for example, the benefit of hourly glucose checks to measure *GV* if an ultrashort insulin preparation is used for SH (particularly, if severe IR is present).

SQ regular insulin consistently lowered *MGC* and *GV* in a linear fashion, thus making the preparation better suited for a sliding-scale protocol. In terms of *GV* and hypoglycemia, the inferior performance of SQ Lispro insulin was a result of its shorter duration of action; thus, SQ Lispro insulin may not be the best choice for patients who are continually postprandial.

Clinical trials are needed to examine whether these theoretical results represent the glucose-insulin dynamics that occur in intensive care units. If such dynamics are present, their clinical effects should be evaluated.

Finally, patients with severe *IR* were the most prone to an increase in *GV* from a change in exogenous insulin concentrations. Severe *IR* may be an indicator of the underlying-glucose-dynamics’ sensitivity to perturbations inherent in some insulin therapies, particularly to the ultrashort SQ insulin analogs in the background of continuous enteral feedings.

## Abbreviations

SH: stress hyperglycemia
IR: insulin resistance
ID: insulin deficiency
GV: glucose variability
TGC: tight glycemic control
SQ: subcutaneous
MGC: mean glucose concentration
IC: insulin clearance
IC_50_: borderline insulin clearance increased by 50 %
ID: _*p*_: insulin deficiency from decreased pancreatic insulin secretion
